# Relationship between pain, functionality, and body composition in patients with fibromyalgia: cross-sectional study

**DOI:** 10.3389/fmed.2025.1745951

**Published:** 2026-01-12

**Authors:** Edurne Úbeda-D’Ocasar, Noemí Mayoral-Gonzalo, Blanca Pedauyé-Rueda, Ariadna Daguerre-Garrido, Cristina Ojedo-Martín, María Jesús Fernández-Aceñero, Juan Pablo Hervás-Pérez, Eduardo Cimadevilla-Fernández-Pola

**Affiliations:** 1 Physiotherapy and Health Research Group (FYSA), Faculty of Health Sciences-HM Hospitals, University Camilo José Cela Urb, Madrid, Spain; 2 Insituto de Investigación Sanitaria HM Hospitales, Madrid, Spain; 3 Department of Legal Medicine, Psychiatry and Surgical Pathology, Complutense University of Madrid, Madrid, Spain; 4 San Carlos Clinical Research Foundation (IDiSCC), San Carlos Clinical Hospital, Madrid, Spain; 5 Department of Chemistry in Pharmaceutical Sciences, Analytical Chemistry Unit, Faculty of Pharmacy, Complutense University of Madrid, Madrid, Spain; 6 Department of Surgical Pathology, Hospital Clínico San Carlos, Madrid, Spain

**Keywords:** fibromyalgia, physical functionality, chronic pain, body composition, anxiety and depression

## Abstract

**Background:**

Fibromyalgia (FM) is a chronic disorder characterised by widespread pain, central sensitisation, and significant psychosocial burden. Patients often present impaired functional capacity, heightened pain perception, and high rates of anxiety and depression. However, the interplay among physical function, psychological distress, and body composition remains insufficiently clarified.

**Materials and methods:**

This cross-sectional study included 80 women with clinically diagnosed FM, aged 18–75 years, recruited from the Afinsyfacro Association (Madrid, Spain). Assessments comprised sociodemographic and clinical variables, functional tests [5-Sit-to-Stand test (5-STST), handgrip strength (HGS), Timed Up and Go (TUG), and 4-m gait speed], algometry, bioimpedance, and validated questionnaires (FIQ, HADS, PSQI, VAS). Correlations between pain, fibromyalgia impact, psychological symptoms, physical performance, and body composition were analysed using Pearson’s or Spearman’s coefficients, as appropriate.

**Results:**

Participants reported severe pain (VAS = 7.03 ± 1.94) and a high disease impact (FIQ = 65.27 ± 16.07). The scores obtained on the HADS-A and HADS-D questionnaires were 12.65 ± 4.56 and 9.93 ± 4.56, respectively. Moderate correlations were observed between depression and both the 5-STST (r = 0.325, *p* < 0.01) and TUG (r = 0.346, *p* < 0.01), while fibromyalgia impact correlated with all functional measures except HGS. Pain correlated with both anxiety (*r* = 0.477, *p* < 0.01) and depression (*r* = 0.430, *p* < 0.01). No significant associations were found between body composition variables and FM impact or pain. The overall fit was significant, *F*(4, 63) = 23.169, *p* < 0.001. Pain (VAS) and depressive symptoms (HADS-depression) contributed independently to higher FIQ.

**Conclusion:**

Effective management of FM requires a multidisciplinary approach integrating therapeutic exercise, pain management, psychological support, and strategies to optimise body composition and overall health. This holistic perspective may reduce symptom burden and improve quality of life in affected individuals.

## 1 Introduction

Fibromyalgia (FM) is a chronic syndrome characterised by widespread musculoskeletal pain, frequently accompanied by fatigue, sleep disturbances, cognitive impairment, and memory deficits (1). In Spain, FM affects approximately 2–4% of the population and constitutes the leading cause of chronic widespread musculoskeletal pain, with women disproportionately affected (2). Despite advances in research and refinement of diagnostic criteria, its incidence continues to rise, underscoring the magnitude of the condition as a persistent health challenge (3). Given its high prevalence and the substantial work absenteeism it generates, FM represents a pressing public health concern (4).

The aetiology of FM remains largely unknown, which poses significant challenges for both accurate diagnosis and the development of effective therapeutic strategies (5). Current management typically involves a combination of pharmacological and non-pharmacological interventions, with the latter gaining increasing importance due to their potential to improve quality of life and alleviate pain while avoiding the adverse effects commonly associated with pharmacotherapy (6).

Mounting evidence suggests that FM is underpinned by alterations in central pain processing. Functional neuroimaging studies have demonstrated abnormal connectivity patterns, including increased coupling between somatosensory regions and pain-related cortical areas, alongside reduced functional integration within descending inhibitory networks. These disruptions contribute to augmented pain sensitivity and a diminished capacity to modulate nociceptive input, thereby perpetuating chronic pain (7, 8).

In addition to central mechanisms, peripheral factors such as body mass index (BMI) and body fat distribution have been increasingly recognised as relevant modulators of symptom severity in FM (9). Another peripheral mechanism related to the development of FM is small fibre pathology, which affects approximately 50% of people with FM (10). Recent evidence indicates that obesity can act as a peripheral disruptor of cortical inhibitory mechanisms, thereby exacerbating clinical manifestations including pain, fatigue and depressive symptoms (11). Moreover, the prevalence of overweight and obesity among people with FM is substantially higher than in the general population, and these conditions are associated with greater tender-point sensitivity, heightened pain perception and poorer physical function (12). Central adiposity and reduced skeletal muscle mass may further compromise mobility and attenuate treatment response. In addition, obesity and higher BMI have been linked to impaired cognitive performance, which may further increase the disease burden in FM patient (13). Taken together, these findings underscore the importance of assessing BMI and body composition in FM patients to better characterise their contribution to disease severity and to inform more personalised management strategies.

The assessment of FM remains challenging due to its heterogeneous clinical presentation, reliance on subjective symptoms, and the absence of specific biomarkers (14). Furthermore, frequent psychiatric comorbidities, particularly anxiety and depression, exert a significant influence on pain perception and functional outcomes (15). As most assessment tools rely heavily on self-report measures, integrating both subjective and objective parameters is essential for achieving a more accurate and personalised evaluation, thereby facilitating improved diagnostic precision and more effective therapeutic planning (6).

The primary objective of the present study is to investigate the relationship between pain, physical capacity and body composition in patients with FM. A secondary objective is to explore the impact of FM on psychological wellbeing, with particular emphasis on anxiety and depression.

## 2 Materials and methods

### 2.1 Participants

The study sample comprised 80 patients diagnosed with FFM according to the 2026 American College of Rheumatology (ACR) criteria. Participants were between 18 and 75 years old, and were members of the Afinsyfacro Association in Móstoles R (Madrid, Spain). All individuals who met the inclusion criteria and voluntarily agreed to participate were enrolled. The research was conducted between 31 October 2024 and 3 May 2025.

**Figure 1:**
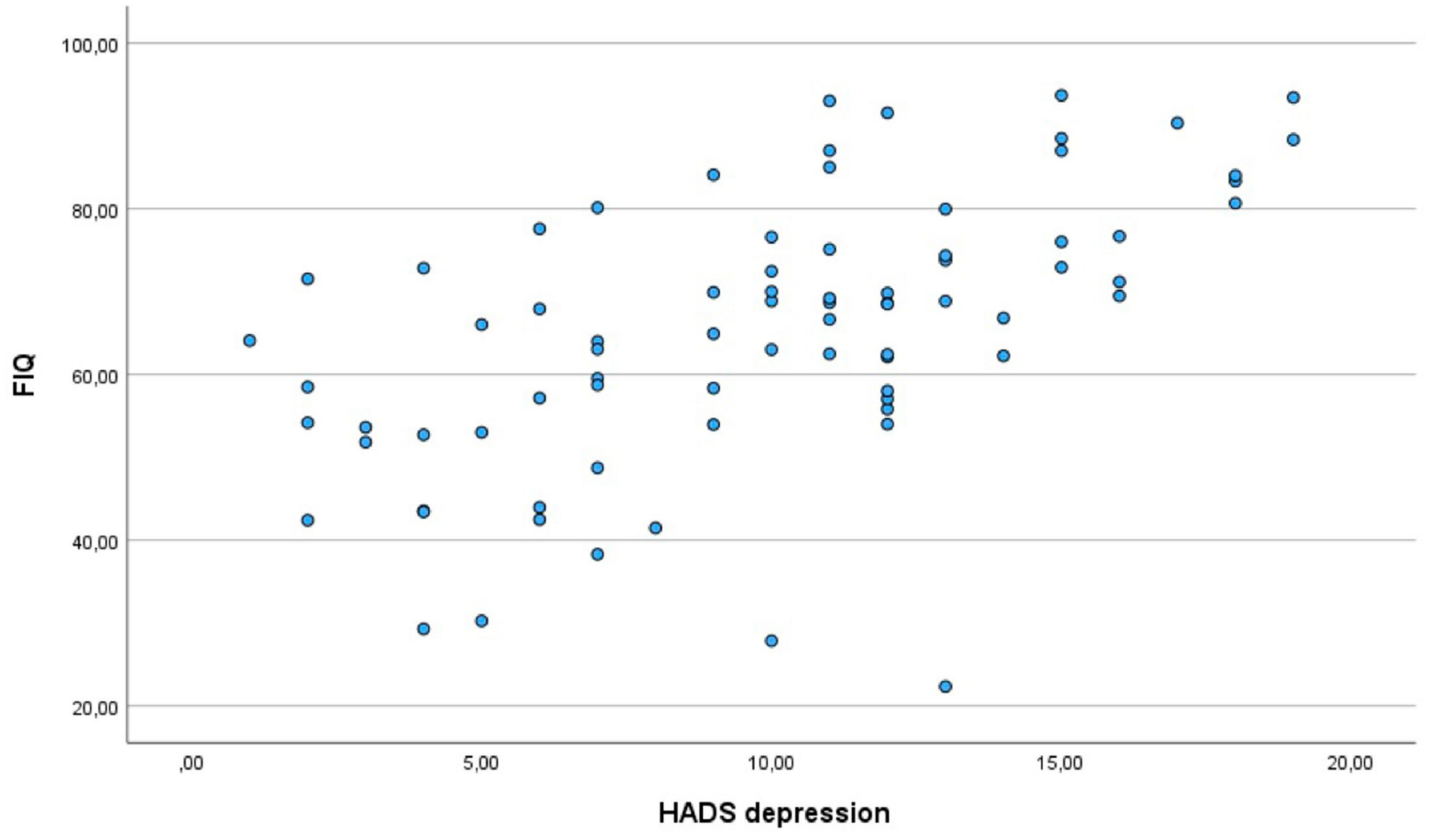
Correlation between depression and the impact of fibromyalgia.

**Figure 2:**
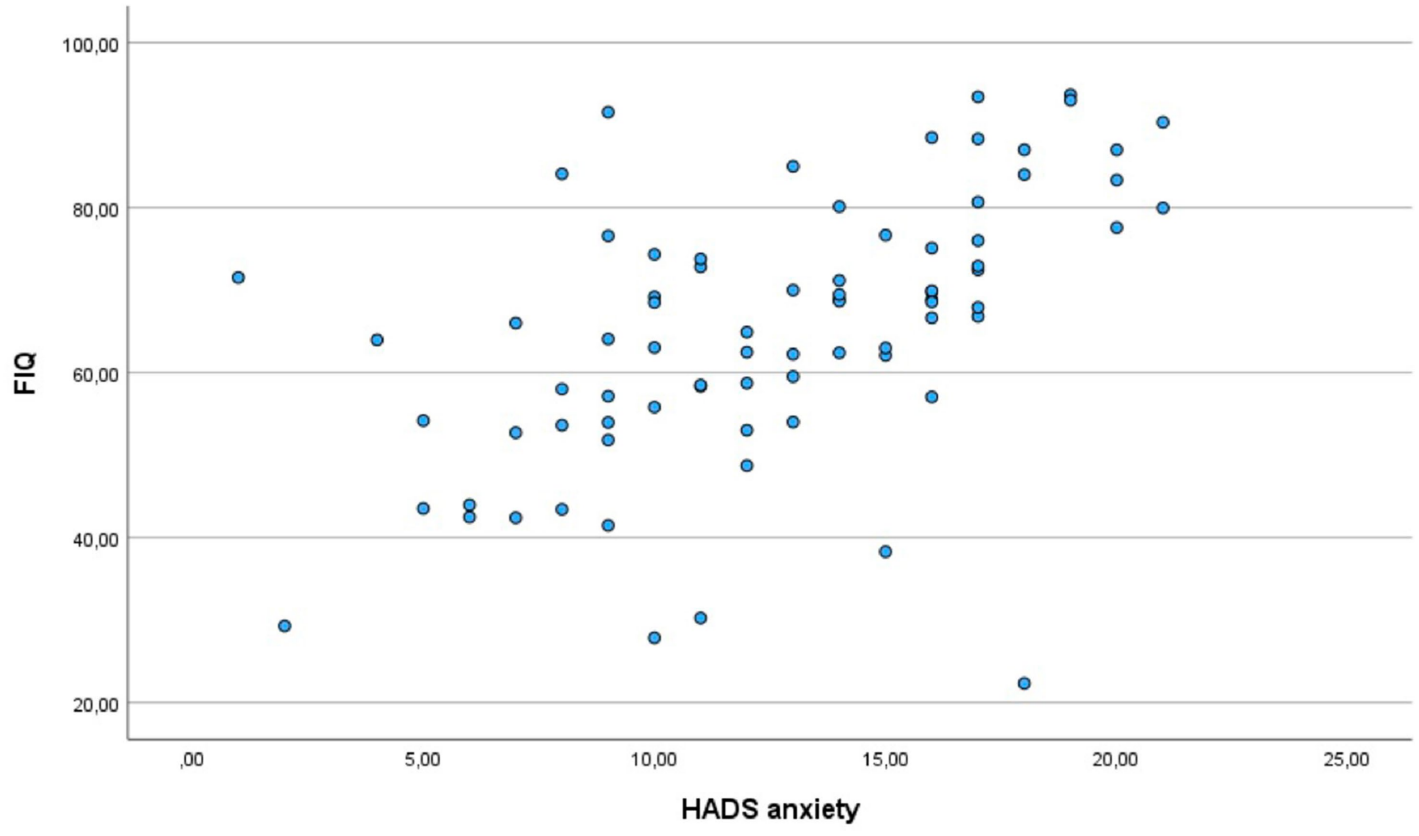
Correlation between anxiety and the impact of fibromyalgia.

The inclusion criteria were as follows: (a) adults (men or women) with a confirmed medical diagnosis of FM; (b) aged between 18 and 75 years; (c) sufficient cognitive ability and comprehension to complete the self-administered questionnaires (Figures 1, 2).

The exclusion criteria were: (i) the presence of other rheumatic or neuromuscular disorders that could interfere with assessment (e.g., rheumatoid arthritis, systemic lupus erythematosus, multiple sclerosis, myopathies); (ii) inability to perform functional assessments; (iii) pregnancy at the time of the study; (iv) recent surgical procedures or traumatic injuries (within the preceding 6 months) that could impair mobility or alter physical functioning; (v) neurological conditions affecting mobility and pain perception (e.g., stroke, multiple sclerosis, Parkinson’s disease, peripheral neuropathies); (vi) metabolic disorders with potential influence on body composition; (vii) current use of pharmacological treatments that could substantially affect neuromuscular function or pain perception.

### 2.2 Measurements

Sociodemographic data collected encompassed age, sex, marital status, educational attainment, employment status, menstrual status, smoking habits, alcohol consumption, and level of physical activity. In addition, clinical variables including body mass index (BMI), presence of comorbidities, use of analgesic and other regular medications, as well as symptoms of anxiety and depression, were recorded.

### 2.3 Functional tests

Five-Sit-to-Stand test (5-STST): Participants commenced seated on a standardised chair (46 cm in height) with feet flat on the floor and arms crossed over the chest. They were instructed to stand fully and sit down five times consecutively as quickly as possible without using their arms. Timing began at the “go” signal and ceased upon completion of the fifth repetition (16). The 5-STST assesses lower-limb functional strength and has demonstrated good reliability (ICC = 0.81) (17). Clinically, it provides a simple and practical measure of lower-limb function and balance, which are often impaired in women with FM, reflecting both strength and fatigue levels.

Handgrip strength (HGS): Upper-limb muscle strength was measured in accordance with recommendations from the European Society for Clinical and Economic Aspects of Osteoporosis, Osteoarthritis and Musculoskeletal Diseases [ESCEO (16)]. Participants were seated with arms resting on the armrests, holding a digital dynamometer (Saehan DHD-1) with the elbow flexed at 90°, wrist positioned in 0–30° dorsiflexion and 0–15° ulnar deviation. Three maximal efforts of 5 s each were performed with the dominant hand, interspersed with 30-s rest intervals. The mean of the three trials was used for analysis. This test is both valid and highly reliable (ICC = 0.95) (18). HGS provides an objective indicator of overall upper-limb strength and functional capacity, which is clinically relevant for evaluating daily living activities in women with fibromyalgia.

Timed Up and Go (TUG): This fundamental functional mobility test required participants to rise from a 46 cm chair, walk 3 m, turn around a cone, return to the chair, and sit down. Two trials were conducted, with sufficient rest between attempts to allow physiological parameters to return to baseline. The mean of both trials was calculated for analysis. The TUG test is valid and reliable for women with fibromyalgia (ICC = 0.93) (19, 20). Clinically, it is widely used to assess mobility, dynamic balance, and fall risk, which are commonly compromised in this population.

4-FFM: Gait speed was assessed using the 4-metre walk test, which exhibits high reliability (ICC = 0.89–0.99) (21). Following ESCEO guidelines, participants were instructed to walk 4 m “as fast as possible” after the cue “Ready, go.” Time in seconds was recorded, and the fastest of two trials was used for analysis (16). This test provides a practical and sensitive measure of walking ability and functional performance, which are frequently reduced in women with FM due to pain, fatigue, and impaired muscle function.

Fibromyalgia Impact Questionnaire (FIQ): The Spanish version of this multidimensional questionnaire was used to assess the impact of FM on functional capacity and quality of life. Scores range from 0 (best condition) to 100 (worst condition) (22, 23). The FIQ is a reliable measure of disease burden, capturing physical, emotional, and social aspects.

Pain: Pain intensity was evaluated using a Visual Analogue Scale (VAS), a 10 cm line on which participants indicated their perceived pain intensity (24). This widely used tool provides a direct and quantifiable measure of subjective pain experience.

Algometry: Pain sensitisation was assessed with an analogue algometer (Fischer FPK 20) applied to specific tender points (bilateral epicondyle, inner knee, and trochanter). Three measurements were taken at ≥30-s intervals, and the mean value was calculated (25). Algometry allows objective quantification of mechanical pain sensitivity, a hallmark feature of FM.

Circumference measurements: Hip, trochanter, and waist circumferences (at the level of the umbilicus) were measured using a standard tape measure, providing anthropometric data relevant to body composition and central adiposity.

Body composition analysis: Weight and composition were assessed using direct segmental multifrequency bioelectrical impedance analysis (InBody^®^ 770, Bilbao, Spain), applying six frequencies (1, 5, 50, 250, 500, and 1,000 kHz). This technique provides accurate estimates of fat mass, lean mass, and segmental distribution, important for evaluating physical health in women with FM.

Anxiety and depression: Symptoms were measured using the Hospital Anxiety and Depression Scale (HADS-A/HADS-D), comprising 14 items divided into two subscales of seven items each. Scores range from 0 to 7 (normal), 8 to 10 (borderline), and 11 to 21 (clinical case) (15). The HADS is validated for chronic pain populations and serves as screening tool for identifying clinically significant mood disorders.

Sleep quality: Sleep was evaluated using the Pittsburgh Sleep Quality Index (PSQI), a 19-item self-report questionnaire. Scores range from 0 to 21, with higher scores indicating poorer sleep quality. Seven components (0–3 each) are summed to produce a global score (26). The PSQI is clinically relevant as sleep disturbances are highly prevalent in women with FM and significantly impact daily functioning and quality of life.

### 2.4 Ethical issues

The study protocol was reviewed and approved by the Ethics Committee of Hospital Clínico San Carlos (approval number 24/745-EC_X) and was registered on https://clinicaltrials.gov/ under the identifier NCT06253273.

The research was conducted in accordance with Spanish legislation, including Law 41/2002, which governs patient autonomy, rights, and obligations regarding clinical information and documentation, and Organic Law 3/2018 on Personal Data Protection and Digital Rights. These regulations restrict the processing of sensitive personal data, such as ethnic origin, political or religious beliefs, union membership, biometric identifiers, health information, or sexual orientation.

All procedures adhered to the principles outlined in the 2014 Declaration of Helsinki of the World Medical Association concerning medical research involving human subjects. Participants’ privacy and confidentiality were rigorously protected, and written informed consent was obtained from all participants; documentation is securely held by the corresponding author.

### 2.5 Statistical analysis

Data were analysed using SPSS software, version 29.0. Categorical variables are presented as frequencies and percentages, while continuous variables are reported as mean ± standard deviation (SD). The normality of continuous variables was assessed using the Kolmogorov–Smirnov test. Homogeneity of variances was evaluated prior to performing parametric tests.

Associations between variables were examined using Spearman’s rho for non-parametric data and Pearson’s correlation coefficient for parametric data. Where appropriate, multiple comparisons were adjusted to control for Type I error. Statistical significance was set at *p* < 0.05. Outliers were identified and examined for plausibility; extreme values were retained only if they reflected valid measurements. All analyses were conducted according to standard assumptions for the selected statistical tests, ensuring robustness and reproducibility of the results. A multiple linear regression model will be used to analyze the relationship between the variable of interest, FIQ, and the other variables in the study.

## 3 Results

The mean age of participants was 54.06 ± 9.66 years. Sociodemographic characteristics of the study sample are summarised in Table 1.

**Table 1:** Sociodemographic characteristics of participants.

Variable	Mean ± SD/%
Age	54.06 ± 9.66
BMI	27.47 ± 5.66
Marital status (a–d)	56.3%; 22.5%; 20.0%; 1.3%
Employment status (e–j)	22,8; 26,6; 12.7%; 19.0%; 5.1%; 13.9%
Menstrual status (k–n)	22.5%; 20.0%; 53.8%; 3.8%
Smoking (o, p)	20.2%; 78.8%
Alcohol (q–s)	1.3%; 36.3%; 61.3%

a, married; b, separated; c, single; d, widowed; e, unemployed; f, full-time employed; g, part-time employed; h, retired; i, permanent disability; j, on sick leave; k, menstruating; l, I am not a women, I am a women who does not.

Descriptive results regarding functionality, pain sensitivity, and body composition are presented in Table 2.

**Table 2:** Descriptive analysis of functionality, pain sensitivity and body composition.

Variable	Mean ± SD
Handgrip strength (kg)	21.17 ± 8.49
5-STST (s)	12.83 ± 5.70
4-m walk test (s)	1.01 ± 0.56
TUG (s)	9.72 ± 7.10
FIQ (0–100)	65.27 ± 16.07
PSQI (0–21)	14.88 ± 4.29
HADS-anxiety (0–21)	12.65 ± 4.56
HADS-depression (0–21)	9.93 ± 4.56
VAS (0–10)	7.03 ± 1.94
Algometry (kg/cm^2^)	
Epicondyle (left)	1.70 ± 0.70
Epicondyle (right)	1.70 ± 0.75
Trochanter (left)	1.99 ± 0.93
Trochanter (right)	1.90 ± 0.94
Knee (left)	1.86 ± 0.92
Knee (right)	1.88 ± 0.86
BMI (kg/m^2^)	27.47 ± 5.66
TBW (kg)	32.56 ± 4.89
Protein (kg)	8.64 ± 1.31
FBM (kg)	27.49 ± 11.32
FFM (kg)	44.37 ± 6.65
SMM (kg)	24.08 ± 3.96
PBF (%)	37.02 ± 8.34
WHR	0.95 ± 0.07
VFL	13.02 ± 5.07
Hip circumference (cm)	105.69 ± 12.38
Trochanter circumference (cm)	53.38 ± 7.31
Waist circumference (cm)	92.15 ± 13.45

5-STST, 5-Sit-to-Stand test; TUG, Time Up Go; FIQ, Fibromyalgia Impact Questionnaire; HADS, Hospital Anxiety and Depression Scale; VAS, Visual Analogue Scale; BMI, body mass index; TBW, total body water; SMM, skeletal muscle mass; PBF, percentage body fat; FBM, fat body mass; FFM, fat free mass; ant leg; WHR, waist-hip ratio; VFL, visceral fat level.

The mean age of participants was 54.06 ± 9.66 years, with the majority being women (96.3%). The mean BMI was 27.47 ± 5.66 kg/m^2^. Most participants were married (56.3%), while 22.5% were separated, 20.0% single, and 1.3% widowed. In terms of employment status, 49.4% were unemployed or on sick leave, and 31.7% were employed. Regarding menstrual status, 53.8% were menopausal, 22.5% menstruating, 20.0% non-menstruating women, and 3.8% men. One in five participants were smokers, while most reported no alcohol intake.

Functional performance was impaired across all tests: mean handgrip strength was 21.17 ± 8.49 kg, 5-STST averaged 12.83 ± 5.70 s, TUG 9.72 ± 7.10 s, and gait speed 1.01 ± 0.56 s. The FIQ score (65.27 ± 16.07) reflected a high disease burden, while PSQI scores (14.88 ± 4.29) indicated poor sleep quality. Anxiety and depression levels were elevated (HADS-A: 12.65 ± 4.56; HADS-D: 9.93 ± 4.56).

Pain intensity was high (VAS: 7.03 ± 1.94), with pressure pain thresholds below 2.0 kg/cm^2^ at all tender points. Body composition analysis showed increased adiposity (PBF: 37.02 ± 8.34%; VFL: 13.02 ± 5.07) and reduced lean mass (SMM: 24.08 ± 3.96 kg).

Moderate positive correlations were observed between pain and psychological symptoms: FIQ scores correlated with both depression (*r* = 0.430, *p* < 0.05) and anxiety (*r* = 0.477, *p* < 0.01), emphasising the strong association between pain and psychological distress in FM.

With respect to the relationship between functional capacity and psychological or clinical outcomes, moderate correlations were observed between performance on physical tests and levels of depression, as well as the overall impact of FM. Specifically, poorer performance on the 5-STST was associated with higher depression scores and greater disease impact. Similarly, TUG performance correlated with depressive symptoms (Table 3).

**Table 3:** Correlations between functional tests, anxiety and depression, fibromyalgia impact, and pain.

Functional tests	5-STST	TUG	HGS	GS (4 m)
HADS-anxiety	0.180	0.213	0.049	−0.259*
HADS-depression	0.325**	0.346**	−0.046	−0.293
FIQ	0.330**	0.269*	−0.065**	−0.212**
VAS	0.245*	0.271*	−0.142	−0.206

HADS, Hospital Anxiety and Depression Scale; 5-STST, 5-Sit-to-Stand test; TUG, Time Up Go; HGS, handgrip strength; GS, gait speed; VAS, Visual Analogue Scale; FIQ, Fibromyalgia Impact Questionnaire. *p < 0.05; **p < 0.01.

No significant correlations were identified between FM impact or pain and the body composition variables assessed (Table 4).

**Table 4:** Correlations between body composition variables, fibromyalgia impact, and pain.

Body composition variables	FMB	FFM	TBW	VFL	BMI
FIQ	0.008	−0.063	−0.059	0.018	−0.035
VAS	0.043	0.049	0.054	0.064	−0.009

FIQ, Fibromyalgia Impact Questionnaire; VAS, Visual Analogue Scale; FMB, fat body mass; FFM, fat free mass; TBW: total body water; VFL, visceral fat level; BMI, body mass index.

A multiple linear regression model was performed with the Fibromyalgia Impact Questionnaire (FIQ) score as the dependent variable of interest. In the final model, the predictors explained a substantial proportion of the variance, with an adjusted *R*^2^ of 0.595, indicating that approximately 60% of the variability in FIQ scores was accounted for by the included variables. The model accounted for 59.5% of the variance in FIQ (*R*^2^ = 0.595; adjusted *R*^2^ = 0.570; standard error of the estimate = 11.040). The overall fit was significant, *F*(4, 63) = 23.169, *p* < 0.001. Pain (VAS) and depressive symptoms (HADS-depression) contributed independently to higher FIQ, whereas handgrip strength and total body water showed marginal/non-significant effects (Table 5).

**Table 5:** Multivariate linear regression model.

Variable	Unstandardized coefficient β	Standardized β coefficients	*p*-value	Confidence interval
VAS	4.993	0.576	0.000	6.56–3.42
HADS-depression	1.141	0.318	0.001	1.8 to −0.491
Handgrip	0.348	0.186	0.052	0.699 to −0.004
TBW	−0.630	−0.183	0.055	0.015 to −1.275

VAS, Visual Analogue Scale; HADS-D, Hospital Anxiety Depression Scale-Depression; TBW, total body water.

## 4 Discussion

The present study sought to examine interrelationships among FM impact, pain intensity, functional capacity, and psychological symptoms (anxiety and depression) according HADS questionnaire, as well as associations with body composition. Consistent with prior work, our participants displayed elevated pain levels (VAS 7.03 ± 1.94) and a high perceived disease burden (FIQ 65.27 ± 16.07).

In comparative populations, such as people with rheumatoid arthritis, individuals with FM often show worse pain experience, catastrophising, and central sensitisation (27). That study, along with ours, emphasises the importance of addressing pain, functional impairment, and psychosocial factors in tandem. Other authors have demonstrated that pain catastrophising and depressive symptoms are key mediators of disability in FM (28).

The literature indicates that high scores in HADS and kinesiophobia are negatively associated with physical functioning and quality of life in FM, fostering avoidance behaviours that exacerbate chronic pain (29). In our sample, the HADS-A and HADS-D questionnaire scores were 12.65 ± 4.56 and 9.93 ± 4.56, respectively. These data suggested that the patients exhibited a moderate level of anxiety and a mild level of depression. We found moderate correlations between depression and performance on the 5-STST (*r* = 0.325) and TUG (*r* = 0.346). Additionally, FIQ correlated moderately with performance in all physical tests. These findings reinforce the idea that multidimensional treatment combining supervised physical exercise and psychological interventions it’s necessary to improve the outcomes for patients with FM (30).

In relation to body composition parameters, a study conducted on Spanish women with FM showed that 31.5% were obese and 38.5% were overweight (31). This trend also occurs in our study, as the mean BMI value is 27.47 ± 5.66, indicating overweight. This indicates that there is a prevalence of excess adiposity in women with FM, which is related to an increase in inflammatory hormones that can cause increased sensitivity to pain (9). A study conducted with women with FM found an association between waist circumference, BMI, and low levels of physical activity, especially with light physical activity (32).

According to the results of our study, we can observe that pain appeared as the main determinant of functional capacity. This reinforces the role of chronic pain as a limitation of functional capacity in subjects with FM. Similarly, total body water and handgrip strength were related to functional capacity. According to the data obtained, these variables follow a trend suggesting that the functional capacity of people with FM depends mainly on muscle quality and neuromuscular efficiency. Other studies have shown that grip strength is an indicator of functional capacity in women with FM (33). Furthermore, women with FM have a higher percentage of fat mass and greater grip strength than healthy women (34). It has also been observed that people with FM present high levels of depression, which has a negative impact on functional capacity and activities of daily living (35).

Low physical activity is a frequent characteristic in FM populations. Sedentarism may exacerbate metabolic dysfunction and foster central adiposity, thus creating a vicious cycle of pain-inactivity. In line with this, we observed moderate correlations between FIQ and physical test performance, reinforcing the notion that physical deconditioning is intertwined with symptom severity. Meta-analyses and systematic reviews show that both endurance and resistance training can ameliorate FM impact, reduce pain, and improve psychological outcomes (36-39). In particular, resistance exercise has recently shown promising effects on depression and fatigue in women with FM, with clinically meaningful improvements (41). Another trial comparing resistance training of different intensities found that low-intensity regimens yielded greater reductions in depressive symptoms at 4 weeks among FM patients (40). Taken together, these results support the adoption of a multidisciplinary treatment approach targeting body composition, motor function, pain modulation, and mental health (13).

These findings highlight the necessity of adopting a multidisciplinary management strategy that integrates therapeutic exercise, tailored pain management, psychological support, and interventions targeting body composition and overall health. Such an approach may optimise functionality, reduce symptom burden, and improve quality of life in this population.

Some of the limitations of this study are that, as it is a cross-sectional design, it prevents causal inferences between the variables examined. Furthermore, although the inclusion criteria were neutral, our sample is exclusively female. This is due to the etiopathogenesis of the pathology and is consistent with the existing literature, but it limits the generalization of the findings to male patients. The sample size is small, which restricts statistical power and external validity. Finally, the absence of a healthy control group prevents direct comparison of functional and psychological outcomes with normative populations.

In subsequent research phases, efforts will be directed towards recruiting a sex-balanced cohort including healthy controls, which would enable normative benchmarking and more accurate modelling of predictive pathways. Moreover, longitudinal and interventional study designs will be essential to elucidate causal mechanisms and guide the development of optimised, personalised therapeutic protocols for individuals with FM.

## 5 Conclusion

Patients in this study reported severe pain and increased sensitivity to pain, especially in the epicondyles. Strong correlations emerged between higher pain levels, greater disease impact and higher scores in anxiety and depression symptoms. Although skeletal muscle mass appeared preserved, it was not associated with physical performance. Pain intensity and depressive symptoms were the strongest predictors of fibromyalgia impact, while grip strength and total body water showed a lesser contribution.

## Data Availability

The original contributions presented in the study are included in the article/supplementary material, further inquiries can be directed to the corresponding author.
